# The relationship between glutamate, dopamine, and cortical gray matter: A simultaneous PET-MR study

**DOI:** 10.1038/s41380-022-01596-6

**Published:** 2022-05-11

**Authors:** Antoine Rogeau, Giovanna Nordio, Mattia Veronese, Kirsten Brown, Matthew M. Nour, Martin Osugo, Sameer Jauhar, Oliver D. Howes, Robert A. McCutcheon

**Affiliations:** 1 Department of Neuroimaging, Institute of Psychiatry, Psychology & Neuroscience, King’s College London, London, UK; 2 Department of Nuclear Medicine, Lille University Hospitals, Lille, France; 3 Department of Information Engineering, University of Padua, Padua, Italy; 4 Department of Psychosis Studies, Institute of Psychiatry, Psychology & Neuroscience, King’s College London, London, UK; 5 Max Planck UCL Centre for Computational Psychiatry and Ageing Research, University College London, London, UK; 6 Institute of Clinical Sciences, Faculty of Medicine, Imperial College London, London, UK; 7 Psychiatric Imaging Group, MRC London Institute of Medical Sciences, Hammersmith Hospital, Imperial College London, London, UK

**Keywords:** Neuroscience, Schizophrenia

## Abstract

Prefrontal cortex has been shown to regulate striatal dopaminergic function via glutamatergic mechanisms in preclinical studies. Concurrent disruption of these systems is also often seen in neuropsychiatric disease. The simultaneous measurement of striatal dopamine signaling, cortical gray matter, and glutamate levels is therefore of major interest, but has not been previously reported. In the current study, twenty-eight healthy subjects underwent 2 simultaneous [^11^C]-( + )-PHNO PET-MRI scans, once after placebo and once after amphetamine in a double-blind randomized cross-over design, to measure striatal dopamine release, striatal dopamine receptor (D_2/3_R) availability, anterior cingulate glutamate+glutamine (Glx) levels, and cortical gray matter volumes at the same time. Voxel-based morphometry was used to investigate associations between neurochemical measures and gray matter volumes. Whole striatum D_2/3_R availability was positively associated with prefrontal cortex gray matter volume (pFWE corrected = 0.048). This relationship was mainly driven by associative receptor availability (pFWE corrected = 0.023). In addition, an interaction effect was observed between sensorimotor striatum D_2/3_R availability and anterior cingulate Glx, such that in individuals with greater anterior cingulate Glx concentrations, D_2/3_R availability was negatively associated with right frontal cortex gray matter volumes, while a positive D_2/3_R-gray matter association was observed in individuals with lower anterior cingulate Glx levels (pFWE corrected = 0.047). These results are consistent with the hypothesis that the prefrontal cortex is involved in regulation of striatal dopamine function. Furthermore, the observed associations raise the possibility that this regulation may be modulated by anterior cingulate glutamate concentrations.

## Introduction

An improved understanding of the relationship between cortical structure and neurochemistry has the potential to advance our pathophysiological understanding of diseases in which simultaneous disruptions exist [1].

The striatum exhibits a high degree of connectivity to both the cortex and other subcortical structures [2, 3], and a variety of inputs may regulate striatal dopamine release. There is in vivo evidence that local striatal glutamate concentrations positively correlate with dopamine levels and may precede dopamine release [4], but cortical glutamatergic projections to the midbrain also play a role [5, 6]. Preclinical work suggests this latter circuit involves cortical parvalbumin interneurons, and their activation reduces striatal dopamine release, likely by inhibiting excitatory projections to the midbrain [7].

Previous studies in humans have used multimodal neuroimaging to investigate these circuits. Links have been highlighted between frontal gray matter volumes and dopamine, specifically an inverse relationship with dopamine synthesis capacity in people with psychosis [8], an inverse relationship with dopamine release and a direct one with D_2_ receptor availability in healthy subjects [9, 10]. However, these have been examined separately and the interaction with the glutamatergic system has not been previously investigated.

This interaction deserves exploration given the anatomical links between cortex and striatum [11], the inverse relationship between cortical glutamate and striatal dopamine synthesis [12], and their possible combined involvement through corticostriatal pathways in neuropsychiatric disorders such as schizophrenia [5], addiction [13], or depression [14].

We have previously observed that, in individuals with schizophrenia, lower dorsolateral prefrontal cortex (DLPFC) gray matter volumes are associated with increased striatal dopamine synthesis capacity [8]. This is consistent with a model whereby lower gray matter volume reflects fewer synaptic inputs onto cortico-nigral projections, leading to a disinhibition of nigrostriatal dopamine projections and greater striatal dopamine release [15]. In line with the view that neurobiological alterations in schizophrenia are a matter of degree rather than categorical [16], we hypothesized a similar relationship in healthy individuals, albeit the absolute values are different from those in people with schizophrenia. The binding of D_2/3_ receptor ligands is sensitive to the synaptic levels of dopamine [17, 18]. With a D_2/3_ receptor ligand one would therefore expect to see lower DLPFC gray matter volumes associated with lower binding in the striatum, as lower ligand binding can be taken to represent greater synaptic dopamine levels. Of note, in the previous study, the association between DLPFC gray matter volume and striatal dopamine function was not observed in patients who had not responded to treatment, and treatment response has been associated with lower magnetic resonance spectroscopy (MRS) measured glutamate levels compared to treatment non-responsive schizophrenia [19]. We therefore also hypothesized that a similar interaction might exist in healthy individuals whereby individuals with lower glutamate levels would show this directionality, in contrast to those with higher levels.

Dopaminergic function can be assessed in vivo using positron emission tomography (PET). The ligand [^11^C]-( + )-PHNO is able to index striatal D_2/3_ receptor availability, and, in conjunction with amphetamine administration, can measure dopamine release [18, 20]. The combined glutamatergic metabolites, glutamate+glutamine (Glx) can be measured using proton MRS [19], while T1 structural magnetic resonance imaging (MRI) allows for quantification of gray matter volume [21]. The use of simultaneous PET-MRI offers the advantages of eliminating potential chronological confounds, reduced within-subject variability, and improved spatial resolution [22, 23].

In the present study, we set out to investigate relationships between prefrontal cortex (PFC) gray matter volumes, anterior cingulate cortex (ACC) Glx levels, striatal dopamine receptors, and striatal dopamine release using a simultaneous [^11^C]-( + )-PHNO PET-MRI in a sample of healthy participants. Specifically, we hypothesized to find a direct relationship between PFC gray matter volumes and striatal dopamine receptor availability, an inverse relationship between PFC gray matter volumes and amphetamine-induced dopamine release, and these relationships to be modulated by ACC Glx concentrations.

## Methods

The study was approved by the local National Health Service (NHS) Research Ethics Committee (12/LO/1955) and the Administration of Radioactive Substances Advisory Committee. PET data were previously reported in McCutcheon et al. [24], but the relationship with structural MRI and [^1^H]-MRS measures have not been previously reported.

### Participants

Twenty-eight participants were enrolled through online advertising. Inclusion criteria were age 18 or above and ability to give informed consent. Exclusion criteria included contraindications to MRI or PET scanning (pregnancy, breastfeeding, significant prior exposure to ionizing radiation) or amphetamine administration (hypersensitivity, concurrent monoamine oxidase inhibitor therapy, cardiovascular disease, hyperthyroidism), current or previous significant medical co-morbidity, past traumatic brain injury with loss of consciousness, background of neurological condition or psychiatric disorder (including substance use disorder) as determined by medical review and the Structured Clinical Interview for Diagnostic and Statistical Manual of Mental Disorders IV (DSM-IV), and history of psychiatric disorder in first-degree relatives. Information concerning tobacco and cannabis use was collected. Subjects smoking at least one cigarette daily were considered smokers, five subjects had consumed cannabis at least once and only one subject regularly used on a weekly basis. All subjects provided informed written consent.

### Scanning procedure

Participants were asked to attend two imaging sessions separated by a minimum of 3 days, during which simultaneous PET-MRI scans were acquired. One session was performed under placebo condition (101 mg of lactose/sucrose tablets), the other after oral dexamphetamine administration (0.5 mg/kg). Scanning was initiated 3 h after placebo or amphetamine administration [20, 25]. Placebo/amphetamine order was determined by prior randomization. Participants and staff involved in the scan sessions were blind to the condition, the number of administered placebo tablets were matched to the number of amphetamine tablets, and blinding was maintained throughout the scanning sessions. Researchers were also blind to drug status during initial image analysis.

PET-MRI data were acquired simultaneously using a General Electric (Milwaukee, WI, USA) SIGNA 3 Tesla scanner. Dynamic PET data were acquired over 90 min using 26 frames (8 × 15 s, 3 × 60 s, 5 × 120 s, 5 × 300 s, 5 × 600 s), immediately following the administration of a bolus of [^11^C]-( + )-PHNO over 30 s (dose 0.020–0.029 μg/kg; mean mass 1.44 μg, mean injected activity 140 MBq), PHNO is a dopaminergic agonist binding D_3_ receptors and high affinity D_2_ receptors [26]. [^11^C]-( + )-PHNO PET has an excellent reproducibility with intraclass correlation >0.9 for the striatum/pallidum [27, 28], while radiotracer specific binding is sensitive to endogenous dopamine release following amphetamine administration [20].

PET signal at the beginning of the acquisition rapidly changes and is essential for modeling. Heat generated by the MRI gradient coils was noted to slightly affect the PET signal, so to preserve the integrity of the signal MRI scanning was started 10 min after the initiation of PET scanning [29]. Following the initial 10 min period of PET-only scanning, the acquisition continued with simultaneous PET-MRI scanning. An attenuation correction map was generated through zero echo time (ZTE)–based MR with the following settings: flip angle = 0.8°, voxel size = 2.4 mm isotropic, matrix = 110 × 110 × 116, acquisition time = 42 s, number of averages = 4, and bandwidth = ± 62.5 kHz. Reconstruction of dynamic PET images was performed with the Vue Point FX-S (VPFX-S) algorithm based on a 3D Ordered Subset Expectation Maximization (OSEM) algorithm that also includes point spread function modeling and time-of-flight information [30, 31]. It corrected for detector normalization, randoms, scatter, dead time, and radioactive decay with the following parameters: 6 iterations, 16 subsets, no post-reconstruction smoothing, voxel size = 2 × 2 x 2.78 mm, and matrix = 128 × 128 × 89. Finally, the previously generated ZTE map was used for attenuation correction [32].

A 3-dimensional BRAVO T1-weighted structural scan was acquired using the following settings: inversion time (TI) = 400 ms, TE = 3.2 ms, repetition time (TR) = 8.5 ms, flip angle = 12°, 1 mm isotropic voxels, matrix = 256 × 256, and number of slices = 188. To acquire [^1^H]-MRS data, a 20 × 20 x 20 mm voxel was placed midline on the anterior cingulate (ACC) region (Supplementary Fig. S1), 13 mm above the anterior portion of the corpus callosum genu and perpendicular to the anterior commissure–posterior commissure line to maximize gray matter inclusion [33]. Auto prescans were used for shimming and water suppression enhancement. Point RESolved Spectroscopy (PRESS) spectra were obtained via the General Electric PROton Brain Examination (PROBE) sequence including water suppression with the following parameters: TE = 30 ms, TR = 3 s, and 96 averages.

### Image analysis

#### Positron emission tomography

The Molecular Imaging And Kinetic Analysis Toolbox (MIAKAT) version 4.3.13 in MATLAB 8.2 (MathWorks, Natick, MA, USA) was used for PET data analysis [34]. The motion was corrected by registering each frame of the dynamic sequence on a single frame of reference, which was the sixteenth frame acquired between 13–15 min after injection [35]. Each subject’s PET scan was then coregistered to their T1 structural scan.

The Montreal Neurological Institute (MNI) structural template underwent a non-linear transformation to allow co-registration to each subject’s T1 structural scan. An in-house MNI-based atlas including the whole striatum, its limbic, associative, and sensorimotor functional subdivisions (as defined by Martinez et al. [36]) underwent the same transformation. Then, using a simplified reference tissue model (SRTM) and the voxel time activity curves, the nondisplaceable binding potential (BP_ND_) of [^11^C]-( + )-PHNO was estimated at each voxel [37, 38]. The cerebellum was chosen as the reference region due to its low D_2/3_ receptor binding [39], and each participant’s mean BP_ND_ were calculated for each region-of-interest (ROI). D_2/3_ receptor availability at baseline is given by BP_ND_ under placebo condition. Dopamine release (∆BP_ND_) can be estimated by quantifying the change between placebo and amphetamine conditions in proportion to baseline as follows:

$$\Delta BPND = 100 \times \frac{{BPND_{placebo} - BPND_{amphetamine}}}{{BPND_{placebo}}}\%$$

Whole striatal dopamine receptor availability and amphetamine-induced dopamine release were used for the primary analysis, while additional exploratory analyses were conducted for the striatal functional subdivisions.

#### MRS analysis

Glutamate + glutamine (Glx) levels were assessed with an LCModel 6.3-I0 (http://s-provencher.com/lcmodel.shtml). Quality of spectra was inspected and metabolites demonstrating a line width (full width at half maximum) ≤0.1 ppm, Cramér–Rao lower bounds ≤20%, and signal to noise ratio ≥5 were analyzed. The Statistical Parametric Mapping 12 (SPM12; Wellcome Trust Centre for Neuroimaging, London, UK, http://www.fil.ion.ucl.ac.uk/spm) segmentation algorithm was used to identify relative distribution of white, gray matter, and CSF in the ACC voxel. CSF inclusion was corrected for with the following formula where M_corr_ = corrected metabolite value, M = raw metabolite value, WM = white matter, and GM = gray matter:

$$M_{corr} = \frac{{M \times \left( {\left[ {WM + GM} \right] \times 1.22 + \left[ {CSF \times 1.55} \right]} \right)}}{{WM + GM}}$$

### Voxel-based morphometry

Voxel-Based Morphometry (VBM) analysis was performed using SPM12 in MATLAB 8.2. This analysis examined relationships between gray matter volumes and (1) D_2/3_ receptor availability (BP_NDplacebo_), (2) amphetamine-induced dopamine release (∆BP_ND_), (3) ACC Glx concentrations, and (4) the interaction between dopamine and glutamate measures. MR data acquired during the placebo sessions were used for all analyses. All T1 structural scans were checked for artefacts, gross abnormalities or poor quality, and the origin was set to the anterior commissure. Images were segmented into gray, white matter and cerebrospinal fluid using the SPM12 probability maps. The Diffeomorphic Anatomical Registration Through Exponentiated Lie Algebra (DARTEL) pipeline was used to improve intersubject registration, especially of small structures [40], and generated gray matter images were subsequently normalized to MNI space, modulated, resliced (1.5 mm isotropic voxels) and smoothed with a 10 mm full width at half maximum (FWHM). Intracranial volumes (ICV) were calculated in the “Tissue Volume” tab [41].

A separate multiple regression model including the intercept and total ICV as nuisance regressors was fitted for each of the three predictor variables of interest: D_2/3_ receptor availability, dopamine release, and ACC Glx. We fitted two additional general linear models for the interaction analysis. Here, the intercept, total ICV, BP_NDplacebo_ or ΔBP_ND_ and Glx were included as nuisance regressors, and mean-centering was performed to orthogonalize the interaction term against main effects. Additional general linear models including age, tobacco smoking and cannabis use as covariates were computed. Dopamine receptor availability, amphetamine-induced release and ACC Glx analyses were preregistered while interaction analyses were conducted post hoc (supplementary information).

A mask comprising Brodmann Area 9 and 46 was generated from the Wake Forest University (WFU) PickAtlas to constrain the analysis to the prefrontal cortex [42, 43]. This region was a priori selected based on our earlier finding of an interaction between its gray matter volume, striatal dopamine synthesis and treatment response in schizophrenia [8], and the consistent anatomical and functional links between prefrontal cortex and striatum in health and disease [44, 45]. We used a 1-point dilation to generate the mask since without it did not include the entire cortical thickness (Supplementary Fig. S2). Peak-level family-wise error correction (pFWE < 0.05) within this ROI was used to assess significance of the results. Gray matter volume data of peak voxels showing correlation with regressors were extracted using the MarsBar 0.44 toolbox (http://marsbar.sourceforge.net) in SPM12. We used the GraphPad Prism 9 software (https://www.graphpad.com) to draw scatter plots. The MRIcroGL software (https://www.nitrc.org/projects/mricrogl) was used for visualization of results and brain rendering [46]. Exploratory analyses on the whole brain, using glutamate (Glu) and linear regressions between other variables were thereafter performed and can be found in the Supplementary Information (Supplementary Tables S1–3).

## Results

### Subject details

Subject demographics, PET and MRS results are summarized in Table 1. Twenty-five subjects attended both sessions, 2 subjects attended the placebo session only, and 1 subject attended the amphetamine session only. The subject that attended only the amphetamine session was excluded, and a subject for whom PET images were unusable was excluded from all analyses involving PET data (Supplementary Fig. S3).

**Table 1:** Subject demographics, PET and MRS results.

Subjects (*N* = 28)	Mean (SD) or %					
Age (years)	23.8 (4.7)					
Female	59.30%					
Tobacco	10.70%					
Cannabis	17.90%					
**Ethnicity**	**Black African**	**Chinese**	**Indian**		**White**	**Other**
	11.10%	11.10%	22.20%		37.10%	18.50%
Right-handed	88.90%					
	**Placebo [mean (SD)]**			**Amphetamine [mean (SD)]**		
BP_ND_ whole striatum	2.52 (0.29)			2.01 (0.29)		
BP_ND_ sensorimotor striatum	2.59 (0.32)			1.9 (0.28)		
BP_ND_ associative striatum	2.43 (0.29)			2.06 (0.31)		
BP_ND_ limbic striatum	2.95 (0.54)			2.28 (0.42)		
Glx in anterior cingulate cortex	20.84 (3.89)			21.79 (3.5)		
GM proportion in ACC voxel	62.3% (15%)			63.6% (7.2%)		
WM proportion in ACC voxel	15.6% (19.6%)			12.2% (6%)		
CSF proportion in ACC voxel	22% (9.8%)			23.3% (8.5%)		

*ACC* Anterior cingulate cortex, *BP*_*ND*_ Nondisplaceable binding potential, *CSF* Cerebrospinal fluid, *Glx* Glutamate+glutamine, *GM* Gray matter, *SD* Standard deviation, *WM* White matter.

### Relationship between dopamine 2/3 receptor availability and gray matter volume

**Fig. 1:**
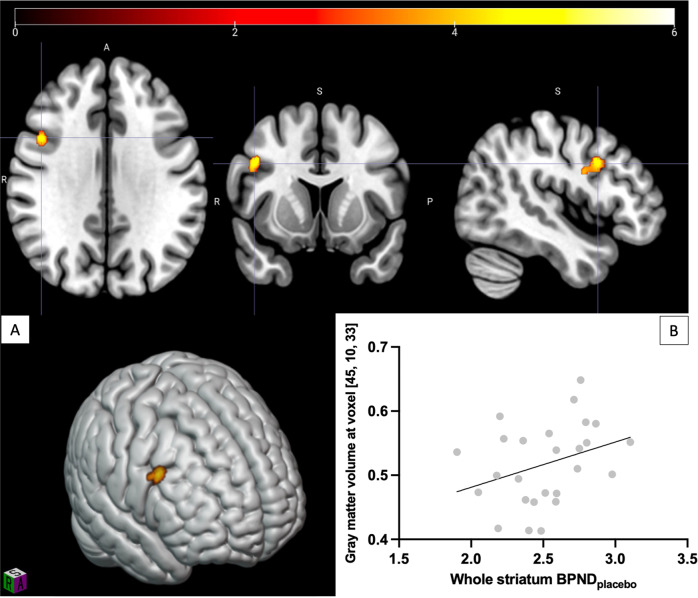
Positive correlation between gray matter volume of a right prefrontal cluster and dopamine receptor availability at baseline in whole striatum. **A** Voxels significant at *p* < 0.05 (FWE corrected) shown, color bar represents T-score. **B** Scatter plot of gray matter density from the significant cluster against whole striatum BP_NDplacebo_.

We found a region (Fig. 1) in the right DLPFC that positively correlated with D_2/3_ receptor availability in the whole striatum (BA9: x = 45, y = 10, z = 33; k = 143, pFWE corrected = 0.048). When repeating the same procedure for the striatal subdivisions (Supplementary Fig. S4), the same region correlated with associative (pFWE = 0.028), but not with sensorimotor or limbic subdivisions. When including age, tobacco and cannabis as covariates, the same association was strengthened with the whole striatal (pFWE = 0.007) and the associative (pFWE = 0.005) D_2/3_ receptor availabilities, and was also observed for the limbic region (pFWE = 0.042).

### Relationship between amphetamine-induced dopamine release and gray matter volume

**Fig. 2:**
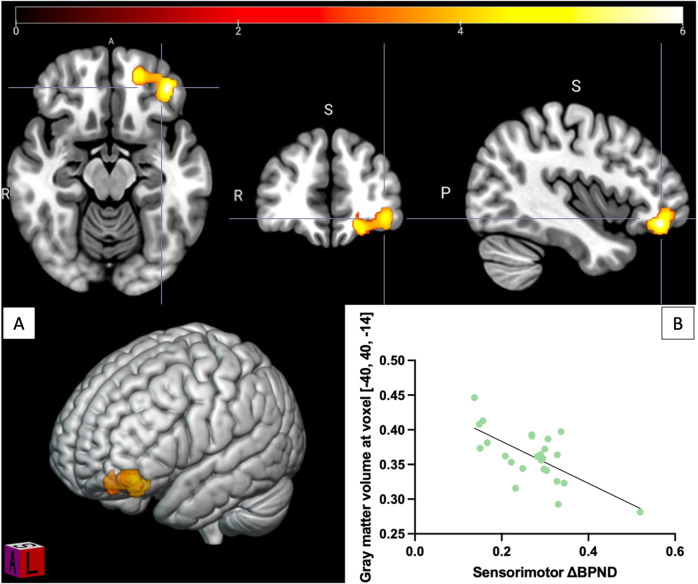
Negative correlation between gray matter volume of a left orbitofrontal cluster and amphetamine-induced dopamine release in sensorimotor striatum. **A** Voxels significant at *p* < 0.05 (FWE corrected) shown, color bar represents T-score. **B** Scatter plot of gray matter density from the significant cluster against sensorimotor striatum ΔBP_ND_.

Binding was significantly lower after amphetamine administration in all ROIs (Supplementary Fig. S5, all *p*-values < 0.0005), reflecting displacement of the radiotracer by endogenous dopamine [17, 47]. Mean whole striatal reduction in ligand binding was 20.4%, standard deviation (SD) = 9.4%. We did not find any regions in the prefrontal cortex that were significantly associated with striatal dopamine release using ΔBP_ND_ as regressor. There was no correlation either when adding age, tobacco and cannabis as covariates. However, in the exploratory analysis, there was a significant inverse relationship between gray matter volume in an orbitofrontal region (Fig. 2) and sensorimotor ΔBP_ND_ (BA47: x = −40, y = 40, z = −14; k = 1462, pFWE corrected = 0.043). This significant relationship was not found when examining whole striatal ΔBP_ND_ (pFWE = 0.16).

### Relationship between anterior cingulate cortex Glx and gray matter volume

Spectroscopic spectra showed mean fit Cramér-Rao lower bounds = 7.1%, SD = 1.7% and range = 5–12%. Mean FWHM was 0.06 ppm, SD = 0.03, and mean signal-to-noise ratio was 12.7 and SD = 3.3. We found a cluster in the left prefrontal cortex that showed a positive association with ACC Glx levels, although only at trend significance (BA46: x = −51, y = 27, z = 22; k = 72, pFWE corrected = 0.065). This trend persisted when using age, tobacco and cannabis as covariates (pFWE = 0.071). We did not find any other prefrontal regions that showed significant association with ACC Glx levels.

### Dopamine receptor and release x Glx interactions

**Fig. 3:**
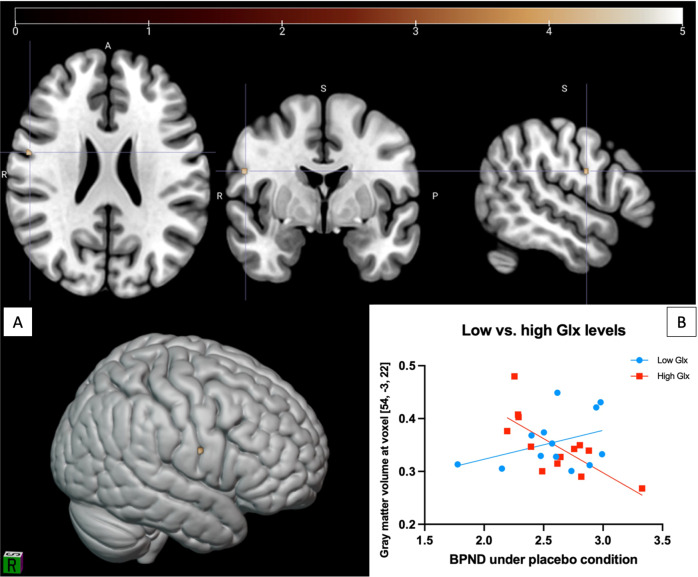
Gray matter volume showing a negative correlation with the interaction term sensorimotor BPNDplacebo:Glx. **A** Voxels significant at *p* *<* 0.05 (FWE corrected) shown, color bar represents T-score. **B** Median splitting was performed to graphically separate subjects with low or high Glx levels. Subjects with low Glx levels show increase in gray matter volume at voxel [54, −3, 22] along increase in sensorimotor receptor availability while subjects with high Glx levels demonstrate the inverse relationship.

A cluster in the right frontal cortex (Fig. 3) showed a significant negative correlation with the interaction term sensorimotor BP_NDplacebo_:Glx (BA6: x = 54, y = −3, z = 22; k = 39, pFWE corrected = 0.047) but not with the whole striatum or other subdivisions. This same correlation persisted using the model including age, tobacco and cannabis as covariates (pFWE = 0.049). For illustration purposes, we used a median split to separate subjects into low vs. high Glx levels, demonstrating that individuals with greater Glx concentrations showed dopamine D_2/3_ receptor availability that was negatively associated with right frontal cortex gray matter volumes, while a positive Glx-gray matter volume association was observed in individuals with lower Glx levels (Fig. 3).

No other gray matter regions showed significant association with the interaction term BP_NDplacebo_:Glx. When testing for the ΔBP_ND_:Glx interaction using the basic or the covariate models, no gray matter clusters showed a significant association.

## Discussion

Our primary findings are that striatal dopamine receptor availability is positively associated with gray matter volumes in the right DLPFC, and evidence for an interaction effect on right frontal cortical gray matter volume between sensorimotor dopamine receptors and ACC Glx levels.

The fact that gray matter volumes most strongly correlated with associative striatum dopamine receptor availability is consistent with the fact that corticostriatal tracts originating from the DLPFC primarily make contact with it [11]. Our findings are in agreement with previous work showing a positive correlation between associative striatum dopamine receptor availability and DLPFC gray matter volume [10]. Interestingly, the cluster identified in the earlier study was in the left DLPFC contrary to the right side in the present study. This inconsistency might be linked to small sample sizes for both studies and bilateral DLPFC volume may be linked to striatal receptors. Supporting this interpretation, a non-significant positive left sided association was observed in the current sample (Supplementary Fig. S6). The main difference in the previous study is the use of [^11^C]-raclopride compared to [^11^C]-( + )-PHNO in the current study. PHNO binds D_3_ receptors in addition to high affinity D_2_ receptors, whereas raclopride binds to both high and low affinity state receptors [48]. The current findings, therefore, suggest that while D_2/3_ high affinity receptors are associated with prefrontal cortex gray matter concentrations, it is unclear if other dopamine receptor subtypes show a similar relationship. There is evidence that PFC projections act to limit striatal dopamine signaling, and if receptor availability is assumed to negatively correlate with synaptic dopamine levels the current findings are consistent with a model in which individuals with lower DLPFC gray matter volumes have reduced inhibition of striatal dopamine signaling. Consistent with this interpretation, lower orbitofrontal gray matter volumes were also associated with greater amphetamine-induced dopamine release in the sensorimotor striatum.

We also found a significant interaction effect between sensorimotor dopamine receptor availability and ACC Glx levels. We therefore show an opposite relationship between dopamine receptor availability and gray matter volume depending on whether subjects have low or high ACC glutamate concentrations (Fig. 3). This finding is of interest given our previous finding of an association between dopamine function and gray matter volumes in individuals with psychosis modulated by treatment response [8]. These two results are potentially consistent, given the evidence that treatment response may relate to glutamate concentrations, in that individuals who respond to antipsychotic have lower ACC Glx concentrations compared to those who do not [49–51]. This raises the possibility that the interaction effect observed in the analysis of D’Ambrosio et al. relating to treatment response may reflect the glutamate levels of those patients, in line with the results of the current study. Finally, the region where we localized the interaction effect includes the premotor cortex which is directly connected to the sensorimotor striatum. Likewise, the ACC also presents connections with the premotor cortex, and there are direct striatum-ACC links [3, 52], illustrating potential anatomical pathways via which this relationship may be mediated.

Although we found a left orbitofrontal volume showing an inverse relationship with amphetamine-induced dopamine release in the sensorimotor striatum, we did not find any significant association for PFC gray matter volumes. In this context, it follows that we did not find any significant interaction between dopamine release and ACC glutamate. We thus failed to replicate a previous finding of an inverse relationship between prefrontal thickness and striatal dopamine release [9], although the methodology between this study and ours varies greatly. Aside from regulation by cortical projections, local cholinergic interneurons contribute to striatal dopaminergic regulation through nicotinic and muscarinic receptors expressed on striatal dopaminergic neurons [53]. Therefore, we speculate that the absence of relationship between dopamine release and prefrontal gray matter volumes suggests the former may be locally regulated by cholinergic interneurons or by other cortical regions while striatal dopamine receptor availability may reflect long-range regulation from the DLPFC.

If confirmed, this could indicate promising avenues for treatment, as it adds to evidence that dysfunction is presynaptic while current treatments primarily target postsynaptic dopamine receptors [54]. Trace amine-associated receptor 1 (TAAR1) agonists and M4 positive allosteric modulators, both could reduce mesostriatal dopamine release and have demonstrated positive results in a phase II trial [55, 56].

### Strengths and limitations

Although the relationship between gray matter volumes and neurochemical systems has been previously studied [8–10, 57, 58], this multimodal study is to our knowledge the first investigating cortex volume in relation to the different aspects of dopaminergic (amphetamine-induced release and receptor availability) and glutamatergic function in the same group of subjects. The study in the same subjects of different components of the dopaminergic neurotransmission and the interaction with the glutamatergic system allows to show that the significant gray matter regions slightly overlap in the dorsolateral prefrontal region (Figs. 1 and 3). This leads to think that a similar study in a larger sample may show further influence of glutamate on the association between striatal dopamine and PFC gray matter volumes. In addition, the use of voxel-based morphometry in SPM12, a free and widespread software, allows for replicability of these findings and robust comparability with other findings based on the same methods.

While the use of [^11^C]-( + )-PHNO BP_ND_ to estimate D_2/3_ receptor availability and dopamine release is well validated, our cross-over design could lead to carry-over effects for subjects who received amphetamine first. A 3-day washout period should, however, allow for good elimination of a product with a 10 h half-life [59]. Despite blinding in the present study, subjects experienced greater subjective drug effects when receiving amphetamine which may have led to some subjects being effectively unblinded [24]. Despite this as analyses were focused on neuroimaging variables rather than subjective report, and volunteers were blind to hypotheses, any inadvertent unblinding is unlikely to have significantly influenced results.

It is not possible with current neuroimaging measures to identify which precise gray matter component underlies the observed relationship between striatal dopamine and gray matter volume. In addition, although participants were screened regarding cannabis use, past substance use may still have been unreported and may have the potential to contribute to the observed findings. While smaller gray matter volumes are often equated to fewer projection neurons, intracortical neuropil, glial cells, interneurons could also contribute to the observed findings. Similarly, [^1^H]-MRS is unable to distinguish between intra- and extra-cellular glutamate limiting the precision of possible inferences [60]. Although we show an interaction between glutamate and dopamine on PFC gray matter volumes, this interaction’s mechanisms are not explored with the present design, and a mediation analysis on a larger sample in a future study may help elucidate them. Finally, these results should still be viewed as preliminary given the moderate sample size and the fact that although stringent multiple comparison correction was employed for each individual analysis, multiple analyses were undertaken.

## Conclusion

In the current study, we demonstrated that the availability of dopamine receptors in the bilateral whole striatum and in the associative subregion positively correlate with right prefrontal gray matter volume, and that there was a negative interactive effect of ACC Glx on a right frontal gray matter volume. These results extend previous findings in schizophrenia and healthy participants to demonstrate interaction across all three systems, with relevance for our understanding of neuropsychiatric disease in which abnormalities are often found to occur simultaneously.

## Supplementary information

Supplementary information

Supplementary Table 1

Supplementary Table 2

Supplementary Table 3

Supplementary Table 4

Supplementary Figure 1

Supplementary Figure 2

Supplementary Figure 3

Supplementary Figure 4

Supplementary Figure 5

Supplementary Figure 6

## Data Availability

Neuroimaging data and analysis scripts are publicly available on GitHub (https://github.com/rogeauA/PFC_GrayMatter_DA-Glx).

## References

[1] Kaminski J, Mascarell-Maricic L, Fukuda Y, Katthagen T, Heinz A, Schlagenhauf F (2021). Glutamate in the dorsolateral prefrontal cortex in patients with schizophrenia: a meta-analysis of (1)H-magnetic resonance spectroscopy studies. Biol Psychiatry.

[2] Calabresi P, Picconi B, Tozzi A, Ghiglieri V, Di, Filippo M (2014). Direct and indirect pathways of basal ganglia: a critical reappraisal. Nat Neurosci.

[3] Peters SK, Dunlop K, Downar J (2016). Cortico-striatal-thalamic loop circuits of the salience network: a central pathway in psychiatric disease and treatment. Front Syst Neurosci.

[4] Lorenz RC, Gleich T, Buchert R, Schlagenhauf F, Kuhn S, Gallinat J (2015). Interactions between glutamate, dopamine, and the neuronal signature of response inhibition in the human striatum. Hum Brain Mapp.

[5] Schwartz TL, Sachdeva S, Stahl SM (2012). Glutamate neurocircuitry: theoretical underpinnings in schizophrenia. Front Pharm.

[6] Karreman M, Moghaddam B (1996). The prefrontal cortex regulates the basal release of dopamine in the limbic striatum: an effect mediated by ventral tegmental area. J Neurochem.

[7] Kokkinou M, Irvine EE, Bonsall DR, Natesan S, Wells LA, Simth M (2021). Reproducing the dopamine pathophysiology of schizophrenia and approaches to ameliorate it: a translational imaging study with ketamine. Mol Psychiatry..

[8] D’Ambrosio E, Jauhar S, Kim S, Veronese M, Rogdaki M, Pepper F (2021). The relationship between grey matter volume and striatal dopamine function in psychosis: a multimodal (18)F-DOPA PET and voxel-based morphometry study. Mol Psychiatry.

[9] Casey KF, Cherkasova MV, Larcher K, Evans AC, Baker GB, Dagher A (2013). Individual differences in frontal cortical thickness correlate with the d-amphetamine-induced striatal dopamine response in humans. J Neurosci.

[10] Kurose S, Kubota M, Takahata K, Yamamoto Y, Fujiwara H, Kimura Y (2021). Relationship between regional gray matter volumes and dopamine D2 receptor and transporter in living human brains. Hum Brain Mapp.

[11] McCutcheon RA, Abi-Dargham A, Howes OD (2019). Schizophrenia, dopamine and the striatum: from biology to symptoms. Trends Neurosci.

[12] Gleich T, Deserno L, Lorenz RC, Boehme R, Pankow A, Buchert R (2015). Prefrontal and striatal glutamate differently relate to striatal dopamine: potential regulatory mechanisms of striatal presynaptic dopamine function?. J Neurosci.

[13] Harada M, Pascoli V, Hiver A, Flakowski J, Luscher C (2021). Corticostriatal activity driving compulsive reward seeking. Biol Psychiatry.

[14] Wu M, Minkowicz S, Dumrongprechachan V, Hamilton P, Kozorovitskiy Y (2021). Ketamine rapidly enhances glutamate-evoked dendritic spinogenesis in medial prefrontal cortex through dopaminergic mechanisms. Biol Psychiatry.

[15] Kim IH, Rossi MA, Aryal DK, Racz B, Kim N, Uezu A (2015). Spine pruning drives antipsychotic-sensitive locomotion via circuit control of striatal dopamine. Nat Neurosci.

[16] Debbane M, Salaminios G, Luyten P, Badoud D, Armando M, Solida Tozzi A (2016). Attachment, neurobiology, and mentalizing along the psychosis continuum. Front Hum Neurosci.

[17] Egerton A, Hirani E, Ahmad R, Turton DR, Brickute D, Rosso L (2010). Further evaluation of the carbon11-labeled D(2/3) agonist PET radiotracer PHNO: reproducibility in tracer characteristics and characterization of extrastriatal binding. Synapse.

[18] Shotbolt P, Tziortzi AC, Searle GE, Colasanti A, van der Aart J, Abanades S (2012). Within-subject comparison of [(11)C]-(+)-PHNO and [(11)C]raclopride sensitivity to acute amphetamine challenge in healthy humans. J Cereb Blood Flow Metab.

[19] McCutcheon RA, Krystal JH, Howes OD (2020). Dopamine and glutamate in schizophrenia: biology, symptoms and treatment. World Psychiatry.

[20] Caravaggio F, Porco N, Kim J, Torres-Carmona E, Brown E, Iwata Y (2021). Measuring amphetamine-induced dopamine release in humans: A comparative meta-analysis of [(11) C]-raclopride and [(11) C]-(+)-PHNO studies. Synapse.

[21] Ashburner J, Friston KJ. Voxel Based Morphometry. In: Squire LR (ed). Encyclopedia of Neuroscience. Academic Press: Oxford, 2009, pp 471–7.

[22] Catana C (2017). Principles of simultaneous PET/MR imaging. Magn Reson Imaging Clin N. Am.

[23] Tahmasian M, Eggers C, Riedl V, Sorg C, Drzezga A (2015). Editorial: utilization of hybrid PET/MR in neuroimaging. Basic Clin Neurosci.

[24] McCutcheon RA, Brown K, Nour MM, Smith SM, Veronese M, Zelaya F (2021). Dopaminergic organization of striatum is linked to cortical activity and brain expression of genes associated with psychiatric illness. Sci Adv.

[25] Asghar SJ, Tanay VA, Baker GB, Greenshaw A, Silverstone PH (2003). Relationship of plasma amphetamine levels to physiological, subjective, cognitive and biochemical measures in healthy volunteers. Hum Psychopharmacol.

[26] Galineau L, Wilson AA, Garcia A, Houle S, Kapur S, Ginovart N (2006). In vivo characterization of the pharmacokinetics and pharmacological properties of [11C]-(+)-PHNO in rats using an intracerebral beta-sensitive system. Synapse.

[27] Lee DE, Gallezot JD, Zheng MQ, Lim K, Ding YS, Huang Y (2013). Test-retest reproducibility of [11C]-(+)-propyl-hexahydro-naphtho-oxazin positron emission tomography using the bolus plus constant infusion paradigm. Mol Imaging.

[28] Gallezot JD, Zheng MQ, Lim K, Lin SF, Labaree D, Matuskey D (2014). Parametric Imaging and Test-Retest Variability of (1)(1)C-(+)-PHNO Binding to D(2)/D(3) Dopamine Receptors in Humans on the High-Resolution Research Tomograph PET Scanner. J Nucl Med.

[29] Vandenberghe S, Marsden PK (2015). PET-MRI: a review of challenges and solutions in the development of integrated multimodality imaging. Phys Med Biol.

[30] Chicheportiche A, Marciano R, Orevi M (2020). Comparison of NEMA characterizations for Discovery MI and Discovery MI-DR TOF PET/CT systems at different sites and with other commercial PET/CT systems. EJNMMI Phys.

[31] Hudson HM, Larkin RS (1994). Accelerated image reconstruction using ordered subsets of projection data. IEEE Trans Med Imaging.

[32] Sousa JM, Appel L, Engstrom M, Papadimitriou S, Nyholm D, Larsson EM (2018). Evaluation of zero-echo-time attenuation correction for integrated PET/MR brain imaging-comparison to head atlas and (68)Ge-transmission-based attenuation correction. EJNMMI Phys.

[33] Jauhar S, McCutcheon R, Borgan F, Veronese M, Nour M, Pepper F (2018). The relationship between cortical glutamate and striatal dopamine in first-episode psychosis: a cross-sectional multimodal PET and magnetic resonance spectroscopy imaging study. Lancet Psychiatry.

[34] Gunn R, Coello C, Searle G (2016). Molecular imaging and kinetic analysis toolbox (MIAKAT) - a quantitative software package for the analysis of PET neuroimaging data. J Nucl Med.

[35] Jiao J, Searle GE, Schnabel JA, Gunn RN (2015). Impact of image-based motion correction on dopamine D3/D2 receptor occupancy-comparison of groupwise and frame-by-frame registration approaches. EJNMMI Phys.

[36] Martinez D, Slifstein M, Broft A, Mawlawi O, Hwang DR, Huang Y (2003). Imaging human mesolimbic dopamine transmission with positron emission tomography. Part II: amphetamine-induced dopamine release in the functional subdivisions of the striatum. J Cereb Blood Flow Metab.

[37] Gunn RN, Lammertsma AA, Hume SP, Cunningham VJ (1997). Parametric imaging of ligand-receptor binding in PET using a simplified reference region model. Neuroimage.

[38] Lammertsma AA, Hume SP (1996). Simplified reference tissue model for PET receptor studies. Neuroimage.

[39] Levant B, Grigoriadis DE, DeSouza EB (1993). [3H]quinpirole binding to putative D2 and D3 dopamine receptors in rat brain and pituitary gland: a quantitative autoradiographic study. J Pharm Exp Ther.

[40] Ashburner J (2007). A fast diffeomorphic image registration algorithm. Neuroimage.

[41] Malone IB, Leung KK, Clegg S, Barnes J, Whitwell JL, Ashburner J (2015). Accurate automatic estimation of total intracranial volume: a nuisance variable with less nuisance. Neuroimage.

[42] Maldjian JA, Laurienti PJ, Kraft RA, Burdette JH (2003). An automated method for neuroanatomic and cytoarchitectonic atlas-based interrogation of fMRI data sets. Neuroimage.

[43] Maldjian JA, Laurienti PJ, Burdette JH (2004). Precentral gyrus discrepancy in electronic versions of the Talairach atlas. Neuroimage.

[44] Haber SN (2016). Corticostriatal circuitry. Dialogues Clin Neurosci.

[45] Fusar-Poli P, Howes OD, Allen P, Broome M, Valli I, Asselin MC (2011). Abnormal prefrontal activation directly related to pre-synaptic striatal dopamine dysfunction in people at clinical high risk for psychosis. Mol Psychiatry.

[46] Rorden C, Brett M (2000). Stereotaxic display of brain lesions. Behav Neurol.

[47] Willeit M, Ginovart N, Graff A, Rusjan P, Vitcu I, Houle S (2008). First human evidence of d-amphetamine induced displacement of a D2/3 agonist radioligand: A [11C]-(+)-PHNO positron emission tomography study. Neuropsychopharmacology.

[48] Graff-Guerrero A, Willeit M, Ginovart N, Mamo D, Mizrahi R, Rusjan P (2008). Brain region binding of the D2/3 agonist [11C]-(+)-PHNO and the D2/3 antagonist [11C]raclopride in healthy humans. Hum Brain Mapp.

[49] Howes O, McCutcheon R, Stone J (2015). Glutamate and dopamine in schizophrenia: an update for the 21st century. J Psychopharmacol.

[50] Howes OD, Kapur S (2014). A neurobiological hypothesis for the classification of schizophrenia: type A (hyperdopaminergic) and type B (normodopaminergic). Br J Psychiatry.

[51] Egerton A, Murphy A, Donocik J, Anton A, Barker GJ, Collier T (2021). Dopamine and glutamate in antipsychotic-responsive compared with antipsychotic-nonresponsive psychosis: a multicenter positron emission tomography and magnetic resonance spectroscopy study (STRATA). Schizophr Bull.

[52] Beckmann M, Johansen-Berg H, Rushworth MF (2009). Connectivity-based parcellation of human cingulate cortex and its relation to functional specialization. J Neurosci.

[53] Cachope R, Cheer JF (2014). Local control of striatal dopamine release. Front Behav Neurosci.

[54] Howes OD, Kambeitz J, Kim E, Stahl D, Slifstein M, Abi-Dargham A (2012). The nature of dopamine dysfunction in schizophrenia and what this means for treatment. Arch Gen Psychiatry.

[55] Brannan SK, Sawchak S, Miller AC, Lieberman JA, Paul SM, Breier A (2021). Muscarinic cholinergic receptor agonist and peripheral antagonist for schizophrenia. N. Engl J Med.

[56] Koblan KS, Kent J, Hopkins SC, Krystal JH, Cheng H, Goldman R (2020). A non-D2-receptor-binding drug for the treatment of schizophrenia. N. Engl J Med.

[57] Jaworska N, Cox SM, Casey KF, Boileau I, Cherkasova M, Larcher K (2017). Is there a relation between novelty seeking, striatal dopamine release and frontal cortical thickness?. PLoS One.

[58] Caravaggio F, Ku Chung J, Plitman E, Boileau I, Gerretsen P, Kim J (2017). The relationship between subcortical brain volume and striatal dopamine D2/3 receptor availability in healthy humans assessed with [(11) C]-raclopride and [(11) C]-(+)-PHNO PET. Hum Brain Mapp.

[59] Sharbaf Shoar N, Marwaha R, Molla M. Dextroamphetamine-amphetamine. StatPearls: Treasure Island (FL), 2021. 29939585

[60] Poels EM, Kegeles LS, Kantrowitz JT, Slifstein M, Javitt DC, Lieberman JA (2014). Imaging glutamate in schizophrenia: review of findings and implications for drug discovery. Mol Psychiatry.

