# Supplementary material for: The relationship between glutamate, dopamine, and cortical gray matter: A simultaneous PET-MR study
Source: Mol Psychiatry. 2022 May 11;27(8):3493–500. doi: 10.1038/s41380-022-01596-6 (PMC9708555; doi:10.1038/s41380-022-01596-6)
Supplement: Supplementary file 1 — Supplementary information [file 41380_2022_1596_MOESM1_ESM.doc]

**SUPPLEMENTARY INFORMATION**

For categorization of tobacco/cannabis, subjects who smoked daily were coded as 1, subjects who smoked less than daily or did not smoke were coded as 0, subjects who regularly used cannabis were coded as 1 and subjects who did not regularly use cannabis were coded as 0.

Prior to the VBM analysis, methods were pre-registered on The Open Science Framework (<https://osf.io/>) and can be found here: [10.17605/OSF.IO/C3BWQ](https://doi.org/10.17605/OSF.IO/C3BWQ). It was decided after methods registration to test for the interaction terms Glx:BP_NDplacebo_ and Glx:∆BP_ND_ therefore they are not mentioned in the study registration. In the same way, the following analyses were performed secondly to registration.

No dilation mask analysis (figure S2):

- whole striatum receptor availability: BA9, x = 45, y = 10, z = 32, pFWE = 0.067
- associative receptor availability: BA9, x = 45, y = 10, z = 32, pFWE = 0.034
- SM BP_ND_:Glx interaction: BA9, x = 54, y = 0, z = 24, pFWE = 0.182

In addition, we performed analyses to explore potential quadratic relationships between Glx, D_2/3_R availability or dopamine release and PFC gray matter volume. The model used included ICV and the regressor in question as covariates of no-interest, and the squared regressor as a covariate of interest. Two regions showed a negative trend with squared sensorimotor BP_NDplacebo_ (x = 56, y = 28, z = 12, pFWE = 0.145; x = 64, y = 2, z = 21; pFWE = 0.261) but not with squared whole striatal BP_NDplacebo_. No other results were observed.
