# Supplementary figures and images for: The relationship between glutamate, dopamine, and cortical gray matter: A simultaneous PET-MR study
Source: Mol Psychiatry. 2022 May 11;27(8):3493–500. doi: 10.1038/s41380-022-01596-6 (PMC9708555; doi:10.1038/s41380-022-01596-6)

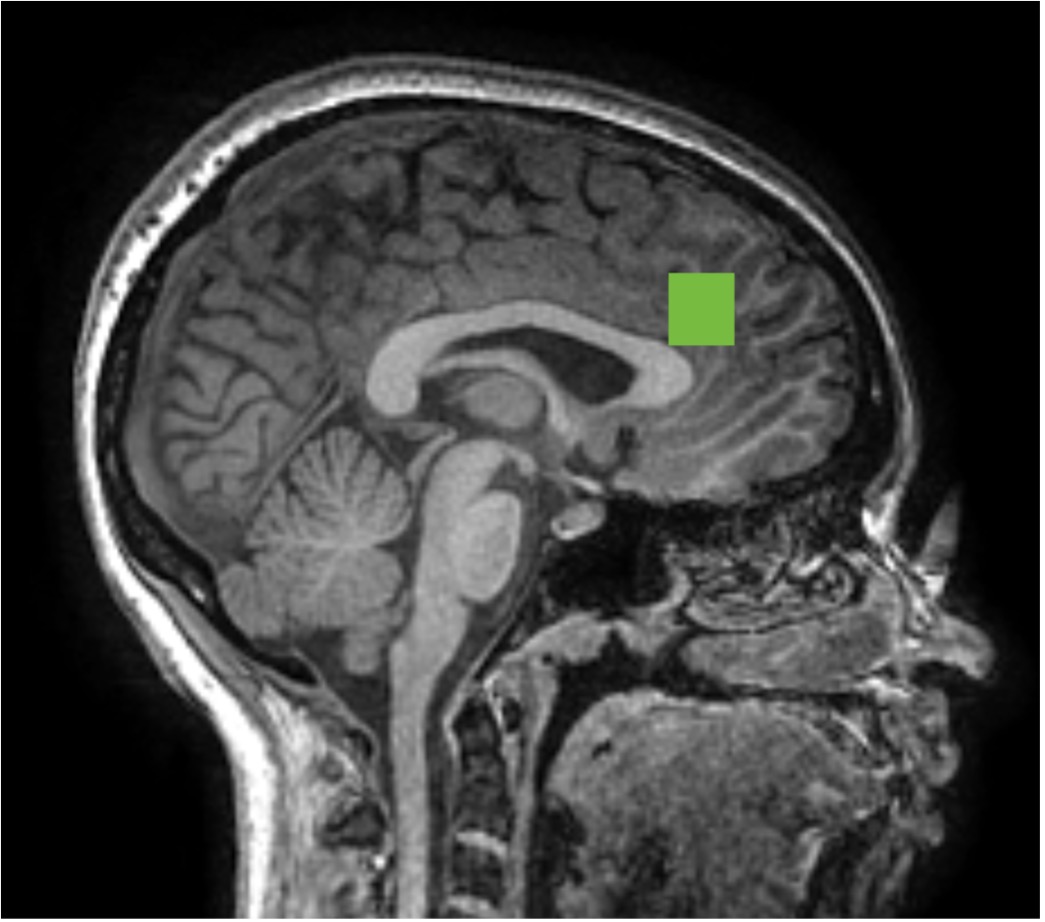

Supplement: Supplementary file 6 — Supplementary Figure 1 [file 41380_2022_1596_MOESM6_ESM.jpg]

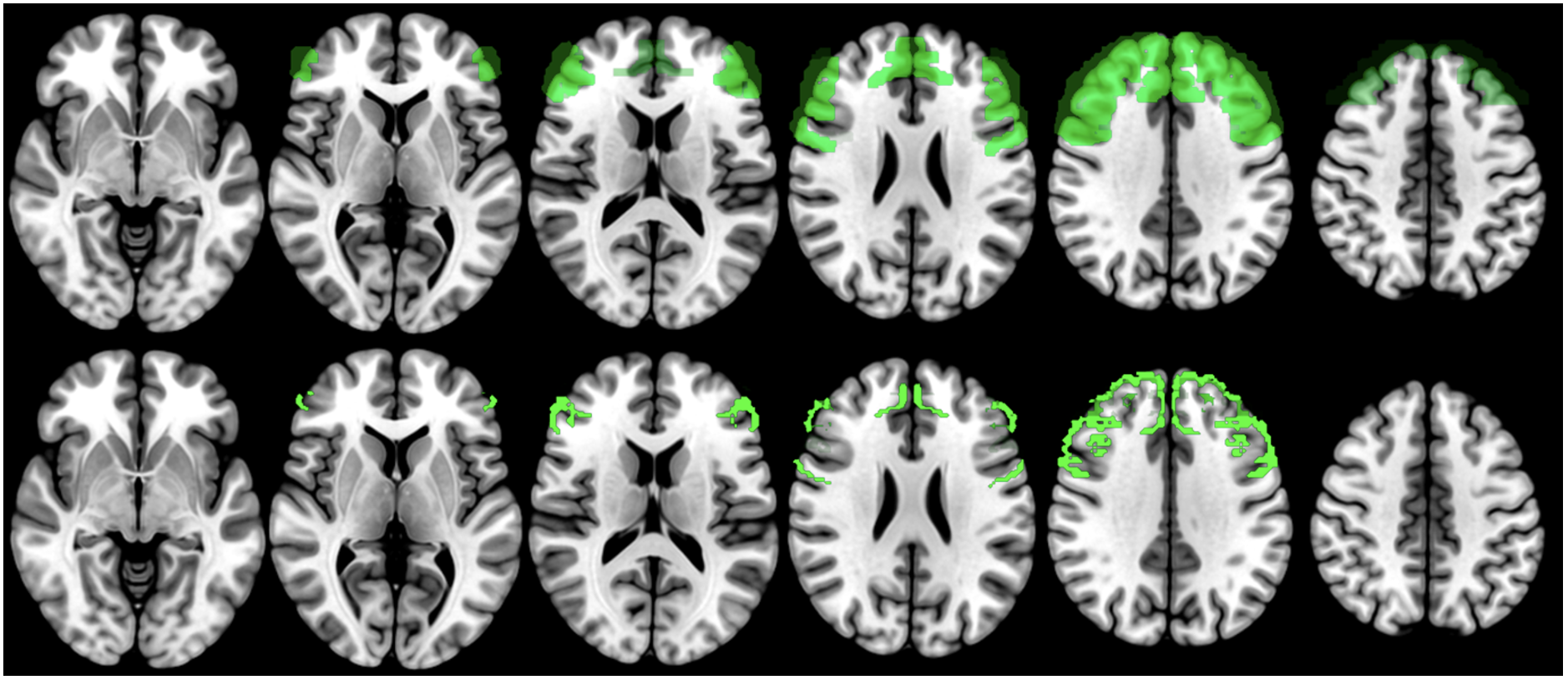

Supplement: Supplementary file 7 — Supplementary Figure 2 [file 41380_2022_1596_MOESM7_ESM.png]

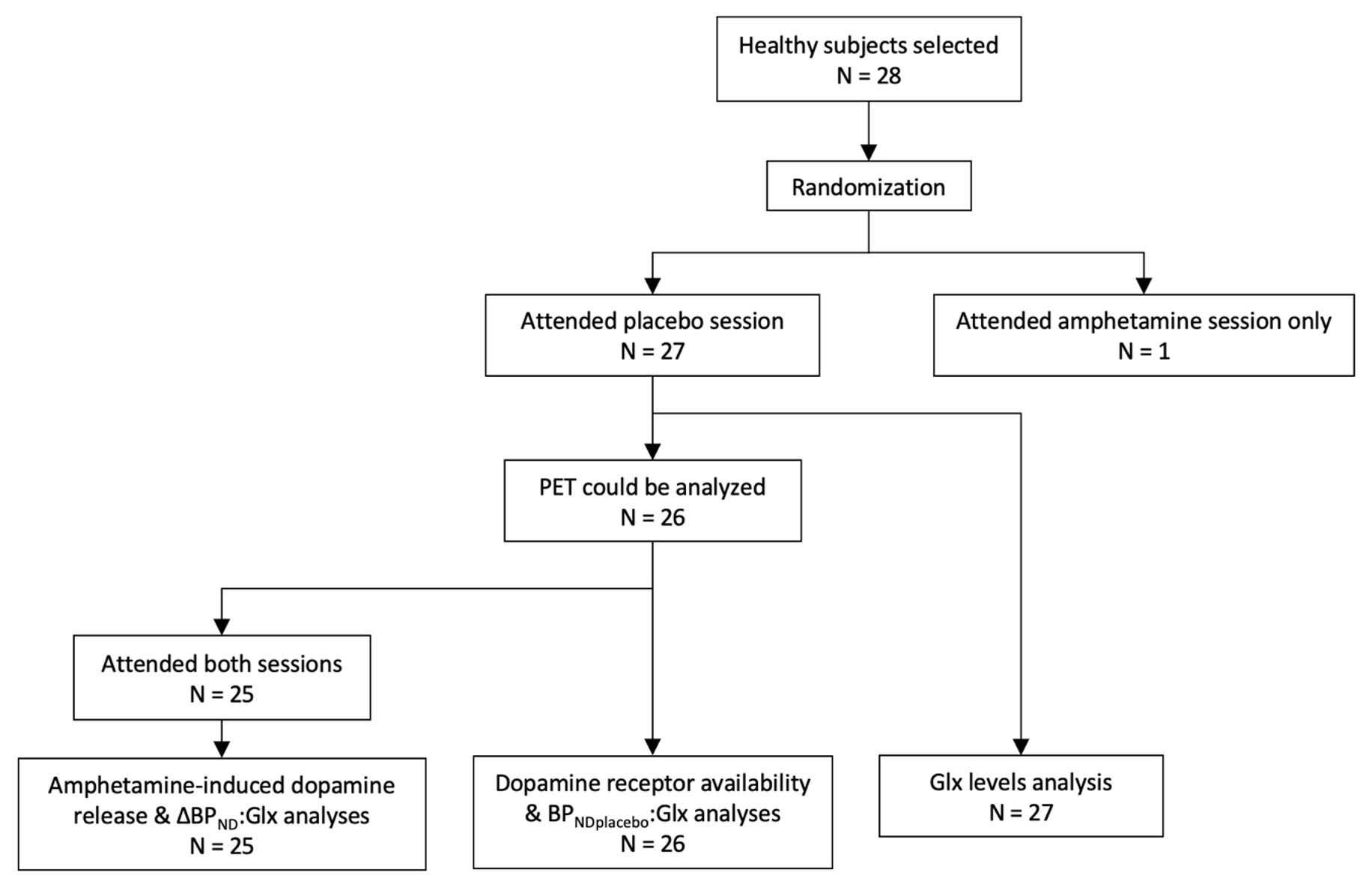

Supplement: Supplementary file 8 — Supplementary Figure 3 [file 41380_2022_1596_MOESM8_ESM.png]

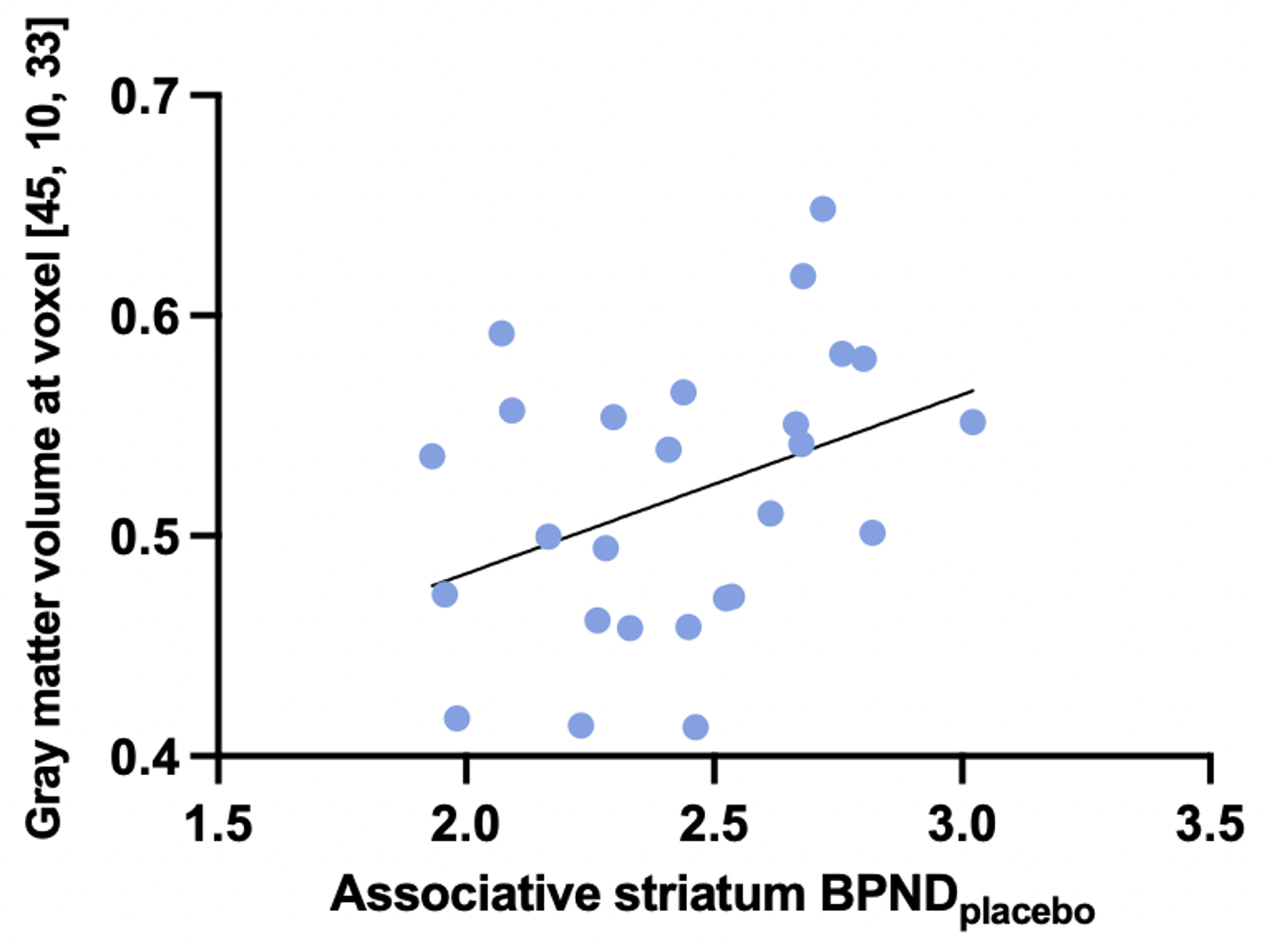

Supplement: Supplementary file 9 — Supplementary Figure 4 [file 41380_2022_1596_MOESM9_ESM.png]

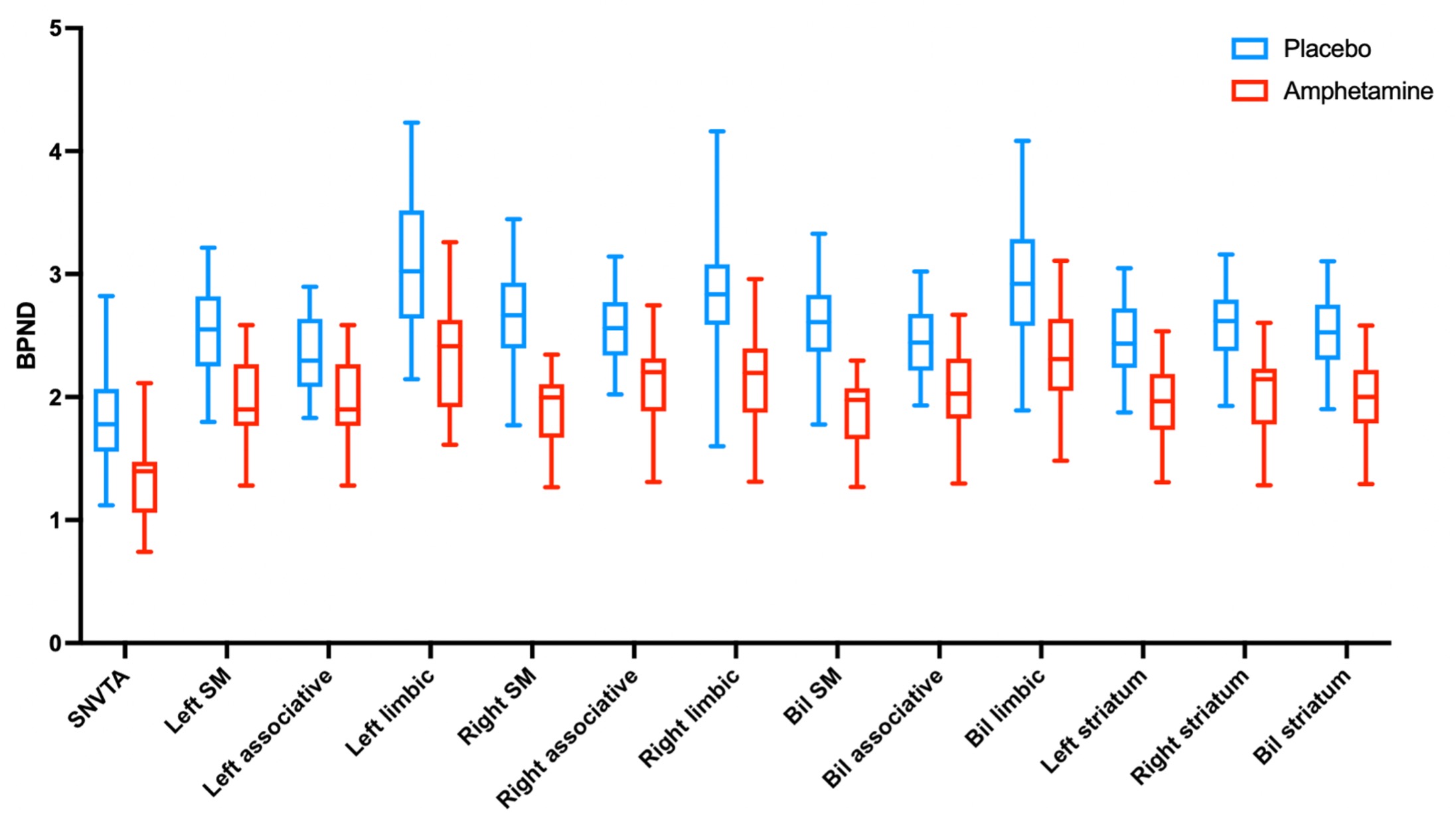

Supplement: Supplementary file 10 — Supplementary Figure 5 [file 41380_2022_1596_MOESM10_ESM.jpg]

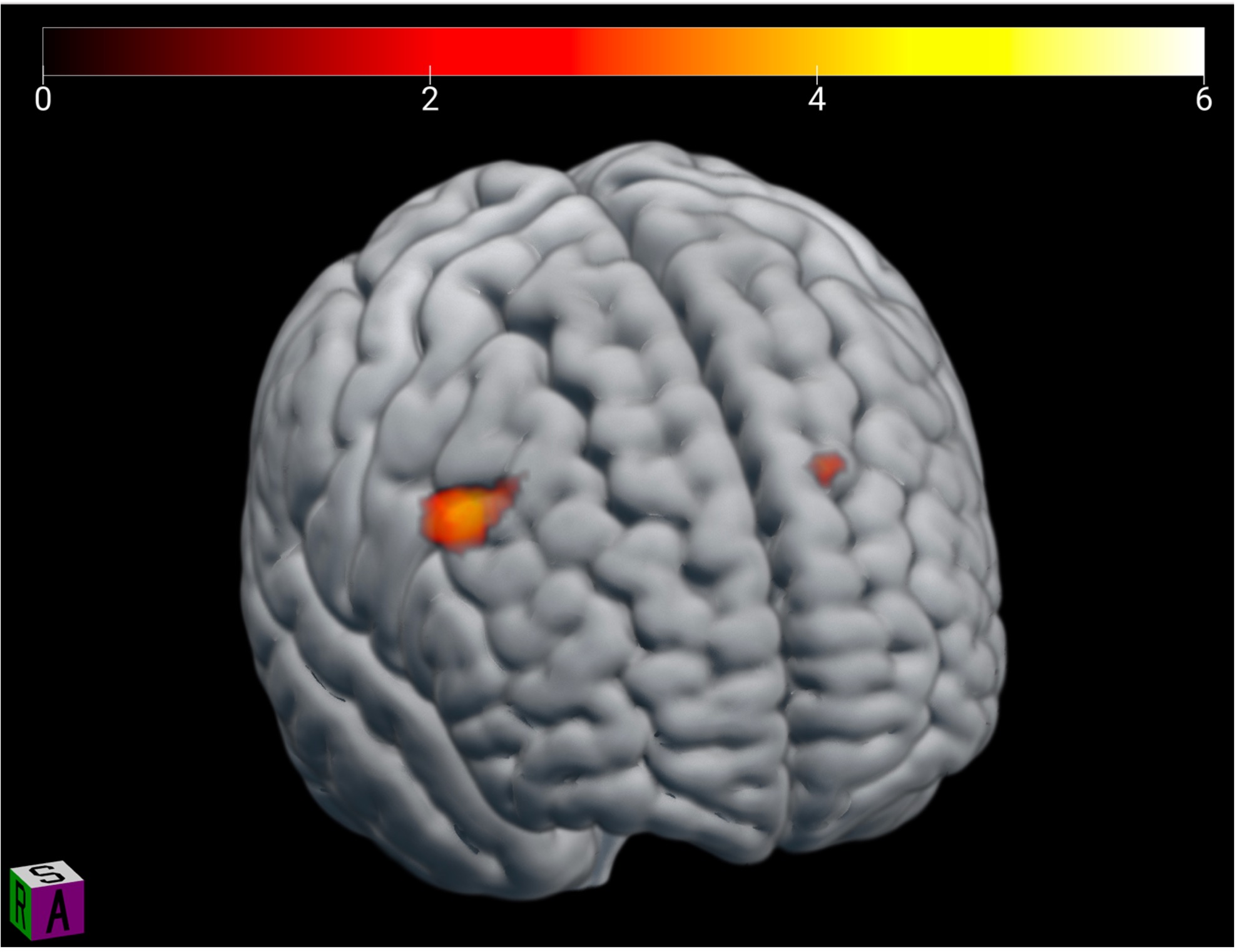

Supplement: Supplementary file 11 — Supplementary Figure 6 [file 41380_2022_1596_MOESM11_ESM.jpg]
